# DRU-Net: Pulmonary Artery Segmentation via Dense Residual U-Network with Hybrid Loss Function

**DOI:** 10.3390/s23125427

**Published:** 2023-06-08

**Authors:** Manahil Zulfiqar, Maciej Stanuch, Marek Wodzinski, Andrzej Skalski

**Affiliations:** 1 Department of Measurement and Electronics, AGH University of Science and Technology, 30-059 Krakow, Poland; zulfiqar@agh.edu.pl (M.Z.); stanuch@agh.edu.pl (M.S.); wodzinski@agh.edu.pl (M.W.); 2 MedApp S.A., 30-150 Krakow, Poland

**Keywords:** pulmonary artery segmentation, DRU-Net, DBCE loss, dense residual blocks

## Abstract

The structure and topology of the pulmonary arteries is crucial to understand, plan, and conduct medical treatment in the thorax area. Due to the complex anatomy of the pulmonary vessels, it is not easy to distinguish between the arteries and veins. The pulmonary arteries have a complex structure with an irregular shape and adjacent tissues, which makes automatic segmentation a challenging task. A deep neural network is required to segment the topological structure of the pulmonary artery. Therefore, in this study, a Dense Residual U-Net with a hybrid loss function is proposed. The network is trained on augmented Computed Tomography volumes to improve the performance of the network and prevent overfitting. Moreover, the hybrid loss function is implemented to improve the performance of the network. The results show an improvement in the Dice and HD95 scores over state-of-the-art techniques. The average scores achieved for the Dice and HD95 scores are 0.8775 and 4.2624 mm, respectively. The proposed method will support physicians in the challenging task of preoperative planning of thoracic surgery, where the correct assessment of the arteries is crucial.

## 1. Introduction

Globally, lung diseases are among the most prevalent and common diseases, which are highly morbid and often fatal. In 2012 [1], the top ten leading causes of death were identified in which chronic obstructive pulmonary disease (COPD), lower respiratory infection, lung cancer, and tuberculosis were ranked 3, 4, 5, and 10, respectively. It has recently been shown that low-dose CT scans for lung cancer screening can reduce mortality [2], which is one of the most prevalent causes of cancer death in both women and men [3]. Furthermore, CT scans are frequently used to detect and monitor lung conditions such as cystic fibrosis, asthma, and interstitial lung diseases. Automated segmentation using CT scans is essential for the development of computer-aided diagnosis, analysis, and to plan surgery. To assist radiologists diagnose lesions, such as pulmonary nodules, the CAD system must accurately identify a region of CT scan which represents the anatomical components of interest. One of the diseases, pulmonary arterial hypertension (PAH), is progressive with a poor prognosis that leads to cardiac arrest [4]. A global estimate suggests that up to 100 million people are affected by this disease [5]. To reduce the risk of death, early identification of the disease and timely treatment are crucial. An accurate interpretation of the structure and geometry is required to diagnose pulmonary hypertension and perform thoracic surgery. Moreover, medical doctors can also plan and modify treatment by observing the segmented pulmonary artery.

A pulmonary artery (PA) originates from the right ventricular chamber [6] and travels through the mediastinum, which contains essential organs such as blood vessels, nerves, a trachea, and the heart. The pulmonary artery is located in the center of the thoracic cavity. The pulmonary artery that follows the aortic arch divides into two parts: the left pulmonary artery and the right pulmonary artery. Furthermore, both the left and right pulmonary arteries are divided into two lobar branches. Afterwards, it divides into segmental and then sub-segmental branches. The branches of the pulmonary tree are extensively subdivided into pulmonary capillaries, which generates a dense web to maximize the surface area for gas exchange in the alveolar wall. Compared to systemic vessels, pulmonary capillaries have thin walls and less smooth muscles; therefore, they are much less resistant to blood flow.

**Figure 1:**
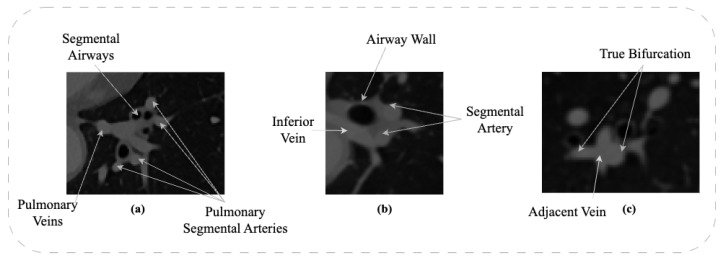
Anatomy of Vessels in CT Data: (**a**) Close Proximity of Arterial and Venous Trees; (**b**) Proximity of Airway Wall to Arterial Tree; (**c**) Vessel Bifurcation.

One of the challenges in segmentation of the pulmonary artery is insufficient contrast between different forms of dense tissues. Figure 1a shows a CT scan of the left lower lobe, which includes segmental venous branches, the airway wall, and segmental pulmonary arteries. Due to the same intensity of veins and arteries, two separate structures appear as a complex blob. Moreover, it becomes difficult to identify the boundaries of the arteries, veins, and airway walls.

Additionally, non-vascular soft tissues, such as lymphatic tissue and airway walls, can also cause segmentation errors. Figure 1b illustrates the geometrical structure of soft tissues. The inferior vein, the segmental airway wall, and the left segmental artery appear as a fused isointensity object. Errors can occur in the segmentation between adjacent tissues.

The geometrical structure of the pulmonary artery further complicates the segmentation process. Figure 1c represents a bifurcation of the left pulmonary artery and the segmental arteries. Likewise, the segmental vein runs through the bifurcation point, which appears to be a part of the parent artery. As a consequence, it is almost impossible to integrate this approach into standard clinical workflow. The structure and topology of the pulmonary arteries are crucial for understanding, planning, and conducting medical treatments in the thorax area. Due to the process of automatic image segmentation, especially using a deep learning framework, the segmentation process could be seamless from the doctor’s point of view.

In recent studies, primarily computed tomography (CT) is used as input volume to perform segmentation [7,8,9]. Manual segmentation of the pulmonary artery is a time-consuming process, so clinicians rely on CT images for preoperative planning of tasks. It is essential to distinguish the pulmonary arteries from the other adjacent structures, which is performed manually by experts and is an exhausting and repetitive process. Furthermore, the use of different visualization techniques, such as 2D visualization, MIPs, or volume rendering, requires additional time for analysis and does not provide conclusive results [10].

Some early approaches to segment the pulmonary arteries were based on semi-automatic techniques, such as feature-based techniques such as active contour, regional growth methods, and gradient vector flow [11]. Zhou et al. [12] enhanced vessels and bifurcation using CT scans by combining the Hessian matrix and the multiscale Gaussian filter. The eigenvalues computed by the Hessian matrix were combined with the response function, which determined the values of vessels and bifurcations while suppressing non-vascular features.

Shikata et al. [13] implemented the vesselness filter to compute the segmentation. The vesselness filter output often leads to disconnected vessels, particularly at bifurcations following thresholding. Seed points were calculated using the output of the vesselness filter. Trajectories were formed using seed points to the nearest junction to connect any disconnected segments.

Lo et al. [14] discussed a technique for segmenting the pulmonary tree using local optimal paths. In the preprocessing stage, the vesselness filter was implemented. The seed points were selected by local maxima of the vesselness filter; a Dijkstra algorithm was implemented around the sphere of the computed seed points. The vessel candidate path was obtained by applying a cost function that was based on vesselness. The optimal path was selected from the candidate paths considering shape, vesselness, and orientation.

In 2012, ref. [15] presented a technique to segment the pulmonary tress by combining optimal local paths with curved planar reformations (CPR). The proposed method implemented a Hessian-based vesselness filter to detect vessels. The detected vessels were straightened by CPR and segmented by applying adaptive thresholding. In the end, Dijkstra’s algorithm was used to find the optimal paths. However, conventional techniques in the field of medicine have only shown a mild success in segmenting complex structures.

Currently, deep learning techniques are implemented to segment complex geometrical structures. Deep neural networks have demonstrated considerable results in several computer vision tasks, such as image segmentation, motion tracking, target recognition, and image classification [16].

Ronnebergerone et al. [17], proposed a U-Net for image segmentation in 2015. Since U-Net has a simple structure, strong generalization capabilities, and strong segmentation abilities, it has been widely studied and implemented in medical image segmentation.

In 2018, Liu et al. [18] proposed the IU-Net architecture, which applied the maximum pooling layers directly to the deconvolutional layers to minimize feature loss and produce improved results. Zhou et al. [19] proposed UNet++, in which skip connections were redesigned, the network showed great results compared to some state-of-the-art backbones.

In 2020, ref. [20] proposed a KiU-Net to segment fine details, it is an over-complete architecture which projects the data to higher dimension in the intermediate layers. Wang et al. [21] implemented a two-stage model to segment the 3D aorta and the pulmonary artery separately based on 3D U-Net. In the first stage, a contrast-enhancing model was built to enhance the contrast of CT images. In the second stage, a neural network model was implemented to segment the aorta and pulmonary artery.

The pulmonary arteries have widespread connections with other complex anatomical structures, such as the pulmonary veins, due to which fine details are often lost during down-sampling. In addition, the structure of the pulmonary artery is also affected by imaging artifacts, variations in contrast, and respiratory motion, leading to a highly diverse dataset to segment. Furthermore, shallow networks are inadequate to segment the complex structure of pulmonary artery and requires a substantial amount of training data. Furthermore, deep neural networks can affect the convergence of the training process, which may potentially lead to vanishing gradient problem. Moreover, the performance of the segmentation model is highly dependent on the selection of the loss function [22], such as the dice loss function, which is implemented to segment structures that comprise a relatively small fraction of the full image [23]. The dice loss function can be unstable in the case of a large difference between the pulmonary artery and the background. To address these limitations, the proposed methodology includes the main innovations:

1. For pulmonary artery segmentation, DRU-Net is proposed, which consists of dense connections, residual blocks, and U-Net structure. The proposed network will address the problem of gradient vanishing and will increase the information flow throughout the network for better segmentation.
2. A hybrid loss function is introduced, which will optimize network performance by stabilizing the loss function and will assist the network in segmenting fine details.

In this study, a combination of dense residual blocks, encoders, and decoders with skip connections is implemented to accurately segment the pulmonary artery. The encoders consists of the combination of residual blocks and down-sampling blocks to extract the features. The decoder comprises a combination of residual blocks and upsampling blocks to reconstruct the segmented output. Each decoder is concatenated with the corresponding encoder to increase the flow of information throughout the network. Moreover, the hybrid loss function is introduced, which combines the properties of the dice loss function and binary cross entropy to stabilize the loss function and optimize the performance of the network for fine details of arteries.

The rest of the paper is structured as follows. Section 2 describes the methods, while Section 3 presents the results. Section 4 includes results-based discussion, and finally Section 5 finishes the research description with conclusions and future work.

## 2. Methods

In this section, the proposed methodology for pulmonary artery segmentation is described. This section provided a description of the dataset, pre-processing, network architecture, and post-processing. Furthermore, the evaluation metrics are also discussed to evaluate the performance of the network.

### 2.1. Dataset Description

To develop a segmentation technique, an open access dataset from MICCAI 2022, Pulmonary Artery Segmentation Challenge (PARSE 2022) is used [24]. The data had been anonymized. The parameters and division of the data set, such as age, pixel spacing, and slice number are described with mean, minimum, and maximum values shown in Table 1.

The dataset was acquired from 3 distinct centers (Chinese PLA General Hospital, Hongqi Hospital affiliated with Mudanjiang Medical University, Harbin Medical University Cancer Hospital). A total of 10 clinical experts with experience of more than 5 years were divided into two teams to annotate the data. Each group included five experts who annotated each scan. The MIMICS software, which is based on the region growing method, was used to perform annotations. At first, the width and level of the window were adjusted to accurately display the PA structure. Then, seed points were obtained manually and iteratively to acquire the coarse mask. Subsequently, experts examined the annotations and fine-tuned the mask. Finally, 5 annotations of each scan were merged by the voting method.

The open access data set contains 100 CT scans which are divided into training, validation, and testing samples with a ratio of 80:10:10. The sizes of the 3D CT scans are between 512 × 512 × 228 to 512 × 512 × 390 while the pixel size is between 0.50 mm/pixels to 0.92 mm/pixels with the slice thickness of 1mm/pixels.

### 2.2. Prepossessing

The dataset is preprocessed to ensure that the network learns the intensity and position of the pulmonary artery accurately. The dataset provided is in NIfTI format. After loading the data set, the following steps are performed:

1. In order to target the lung area, Intensities are clipped to [−800, 500] HU. Due to the nature of CT data, clipping is required. The range of HU values is wider than what can be displayed. The use of clipping ensures that the same structures are assigned similar values after the operation, regardless of the CT scanner on which they were collected. By limiting the intensities to [−800, 500] HU, we focus on the range that is more likely to capture pulmonary vessels and reduce the impact of unrelated structures or artifacts Figure 1.
2. The dataset is normalized between the values [0, 1] for the training of the network.
3. Each sample in the dataset is divided into 9 patches of the size 256 × 256 × number of slices. A patch-based approach is employed as high resolution volumes cannot be processed at their original resolution due to a GPU memory constraint. Furthermore, the patch-based method synthetically generates “more” data by dividing input volumes into patches with varied centers.

**Figure 2:**
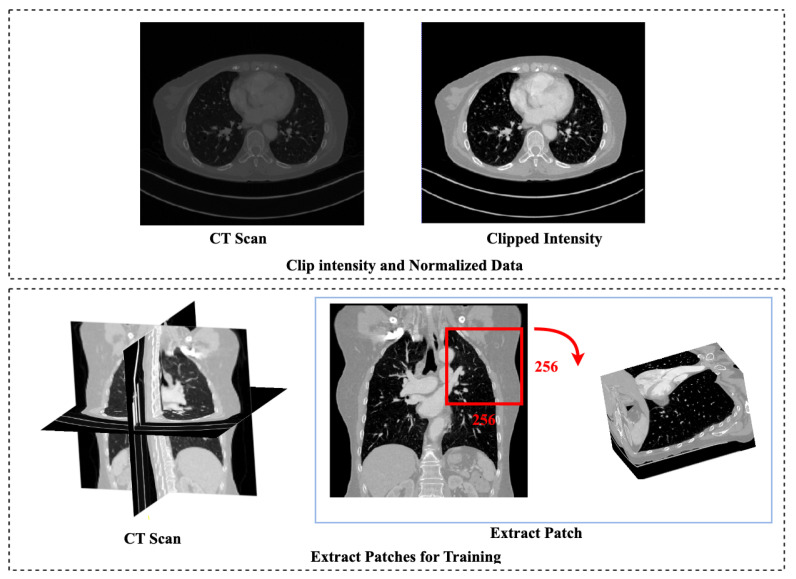
Preprocessing Steps for Pulmonary Artery Segmentation. The first row: Original CT scan and preprocessed Ct scan; The second row: The extracted patch of the size 256 × 256 × number of slices.

After preprocessing, the dataset is saved in the NIfTI format for further processing. Figure 2 illustrates the sequential preprocessing steps that are performed on CT scans of the pulmonary artery before the network training.

### 2.3. Network Architecture

The pulmonary artery has a trunk with thin branches and, in order to segment it, a segmentation network is required to learn the nonlinear transformation of the artery. A shallow network is not able to detect complex structures and requires a huge amount of data for the training process [25].

In the field of medical image segmentation, the U-Net has acquired great results in the case of limited datasets. To learn complex geometry and features, the depth of the neural network is often increased. Deep architectures affect convergence, which can cause vanishing gradients. In this study, dense residual blocks are introduced to solve these limitations. The residual blocks eliminated the gradient disappearance problem, and also provided the fast convergence.

In the residual block, the features are computed by four consecutive units, each containing a 3D convolutional layer with kernel size (3 × 3 × 3) with stride 1, a group normalization layer, and Leaky Rectified Linear Unit (LReLU). Each convolutional layer is operated on the feature maps computed by the previous layer which promotes the feature reuse and improves the overall learning capacity. The group normalization divides the input channels into groups and each group is normalized independently, which enhance the robustness and stability of the training process. Moreover, group normalization (GN) is independent of batch size and its accuracy is stable compared to batch normalization (BN) in a wide range of batch sizes [26]. The Leaky Rectified Linear Unit (LReLU) is introduced as an activation layer. LReLU has a no n-zero slope for negative values, which helps to adopt the non-linear transformation of the features [27], and decrease the risk of dead filters/neurons. The residual dense block also contains a parallel convolutional layer with kernel size (1 × 1 × 1), which is introduced to transform channels before the addition operation. The residual connections take the output of the one block and convert it to the input of the next block and activate it.

**Figure 3:**
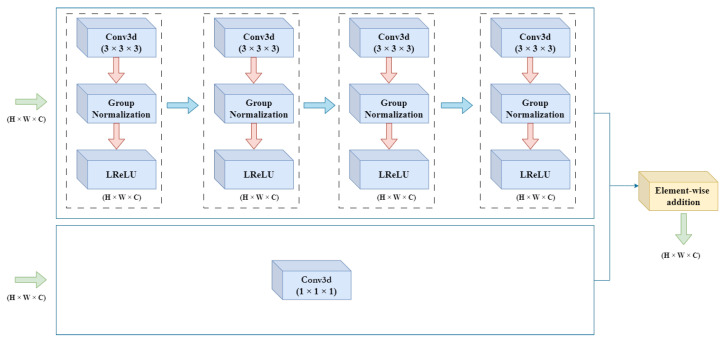
Architecture of Dense Residual Block for the Feature Extraction. The 4 units are connected in series, shown by rectangular dotted boxes, each unit contains a convolutional layer, GN, and LReLU. Furthermore, a parallel convolutional layer is connected with element-wise addition operation for final output of the residual block. The data flow is depicted with arrows.

In this Figure 3, the in-depth visualization of dense residual block architecture is illustrated.

The Dense Residual U-Net is constructed with a four encoder and four decoder blocks to calculate features for the segmentation of the pulmonary artery. To extract the information, the encoder is composed of different combinations of transition down block and residual blocks. The encoders gradually decrease the spatial dimension of the input and increase the number of channels. After each encoder, the spatial dimension is halved, while the number of channels is increased. Progressively, the encoders compute the high-level feature and preserve low-level details. Furthermore, in encoders, a transition down block is introduced instead of max-pooling layers. The maximum pooling layers can lead to information loss since they down-sample feature maps by selecting the maximum values from the neighborhoods. Consequently, a network can be less accurate in segmenting input images. To reconstruct the sample, the decoders are introduced with the combinations of transition up blocks and residual blocks. The decoders gradually increase the spatial dimension of the input while decreasing the number of channels. Each decoder performs an up-sampling operation which is followed by a feature map concatenation of the corresponding encoder block. The decoders reconstruct a high-resolution segmentation, map using the features computed by encoders.

At the end of the network, a 3D convolutional layer of kernel size (1 × 1 × 1) with a sigmoid layer is implemented to calculate the feature map with two categories, background as 0 and segmented artery as 1.

**Figure 4:**
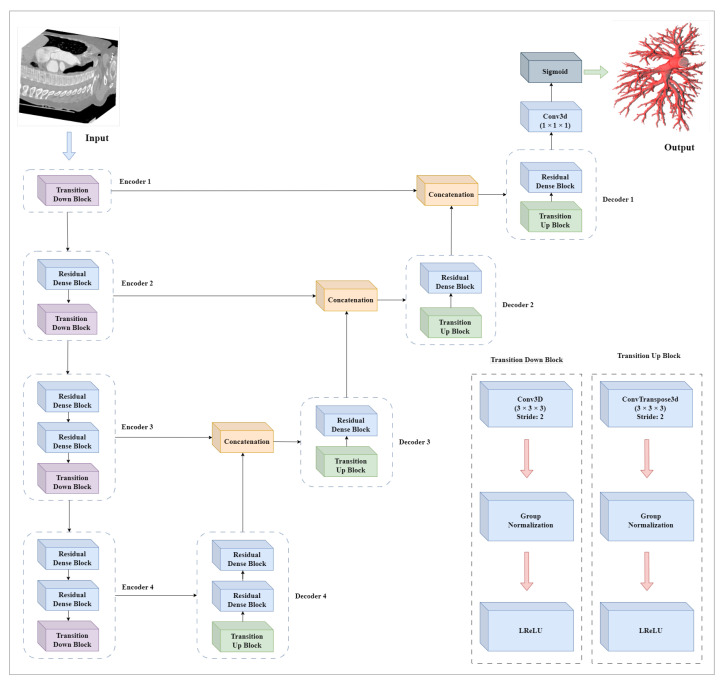
Dense Residual U-Net: A Deep Learning Architecture for Pulmonary Artery Segmentation. Encoders and decoders are illustrated with dotted rectangles containing different combinations of the transition down block, transition up block, and residual blocks. The flow of data in encoders, decoders, and concatenation blocks is described by the arrows.

In this Figure 4, a DRU-Net architecture for the segmentation of the pulmonary artery is described.

#### Hybrid Loss Function

In order to segment pulmonary arteries, a hybrid loss function is proposed that combines the Dice loss function and binary cross-entropy. The hybrid loss function preliminary addresses the challenges such as class imbalance, low contrast between the pulmonary artery and adjacent tissues, and segmenting small size objects. The Dice loss function is effective in resolving class imbalances [28] and is defined as:

(1) $$Loss_{Dice}=1-\frac{2\sum_{i}^{N}p_{i}g_{i}}{\sum_{i}^{N}p_{i}^{2}+\sum_{i}^{N}g_{i}^{2}}$$

where N, $p_{i}$, and $g_{i}$ represent the sum of pixels, ground truth, and predicted label, respectively. During the training process, the Dice loss function can be unstable. If the $p_{i}$, and $g_{i}$ are relatively small compared to the background, the estimated gradient of Dice loss significantly varies.

To stabilize the loss function, the Binary Cross Entropy loss function (BCE) is introduced. The BCE measures the similarity between ground truth and predicted result.

It is defined as:

(2) $$L_{BCE}=-\left(p_{i}log\left(q_{i}\right)+\left(1-p_{i}\right)log\left(1-q_{i}\right)\right)$$

where $p_{i}$ shows the predicted label and $g_{i}$ shows the ground truth.

The combination of a Dice loss function and a binary cross entropy loss function facilitates the network to optimize the learning process with a smooth gradient. The hybrid loss function is defined as:

(3) $$Loss_{DBCE}=\frac{Loss_{BCE}+Loss_{DSC}}{2}.$$

### 2.4. Post-Processing

The computational cost was reduced by dividing each sample into patches during both the training and the testing phases. In the post-processing stage, the predicted patches are reconstructed in their original shape. After segmentation, it is possible that the output may contain false-positive regions. The morphological operations such as, area closing and area opening are used to remove small-size objects.

### 2.5. Network Training and Testing

During the training process, a 10-fold cross-validation (10FCV) was applied. The 10FCV has several advantages that make it more suitable for our study. First, the 10FCV gives the model more data to train on during each iteration, potentially leading to better learning and more robust evaluation. Second, by averaging the results from 10 iterations on a 10FCV, the variance of the estimated performance is typically lower compared to a 5-fold cross-validation. Lower variance implies a more stable estimate of the true performance of the model, reducing the impact of data randomness on the evaluation. Third, the 10FCV allows for a more comprehensive assessment of the model’s ability to generalize to unseen data.

Each CT scan is divided into 9 random patches to create the training and validation set. As a result, 80 CT scans generated 720 patches for training, while 10 CT scans produced 90 patches for validation. To enhance the training process and prevent overfitting, patches are rotated at various angles for augmentation.

For the proposed network, Adam optimizer [29] is implemented with a learning rate of 0.0001, batch size 1, and a total of 200 epochs.

In the prediction phase, 10 CT scans are used, which are pre-processed and converted into patches. The trained network is used to perform segmentation on the testing patches. Following segmentation, post-processing is performed, and the results are compared to the ground truth using evaluation metrics.

**Figure 5:**
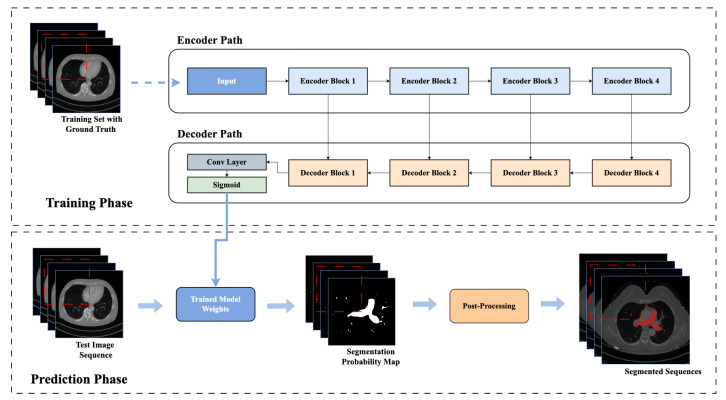
Flow Chart of Network Training and Prediction Phase for Pulmonary Artery Segmentation. In training phase, the network is trained with the annotated training set. In the testing phase, a trained model is used to segment the test image sequence. In the end, the post-processing is applied.

Figure 5 shows the flow chart of the training and prediction phases of the pulmonary artery segmentation.

### 2.6. Evaluation Metrics

To evaluate the performance of the proposed method, the Dice Similarity Coefficient (DSC), and the Hausdorff Distance (HD95) are computed.

The metrics are described as follows:

1. Dice Similarity Coefficient (DSC): The DSC represents the overlap between the predicted results and the ground truth based on Equation (4), where *P* represents the predictions and *G* shows the ground truth.
  (4) $$DSC=\frac{2\left|P\cap G\right|}{\left|P\right|+\left|G\right|}$$
2. 95% Hausdorff distance (HD95): The Hausdorff Distance (HD) is a metric used to compute the maximum distance between two structures.
  (5) $$\begin{array}{cc} HD(X,Y) & =max\left\{d_{XY},d_{YX}\right\}\\ \; & =max\left\{\max_{x\epsilon X}\min_{y\epsilon Y}d\left(x,y\right),\max_{y\epsilon Y}\min_{x\epsilon X}d\left(x,y\right)\right\}. \end{array}$$
  In Equation (5), *X* and *Y* represent the boundaries of two different structures. $d_{XY}$ shows the maximum distance from the calculated minimum distances from the boundary *X* to the boundary *Y*, while $d_{XY}$ represents the maximum distance from the calculated minimum distances from boundary *Y* to boundary *X*. The Hausdorff distance (HD) is extremely sensitive to outliers [30]. As a result, in the field of medical science, HD95 is used. HD95 is the 95th percentile of the Hausdroff Distance. The objective of HD95 is to reduce the impact of a very small subset of outliers.

## 3. Results

To compute the results of proposed methodology, the image dataset is obtained from publicly accessible MICCAI’s Pulmonary Artery Segmentation Challenge, Parse 2022. The results are obtained after a 10FCV on the proposed methodology as shown in Table 2.

**Figure 6:**
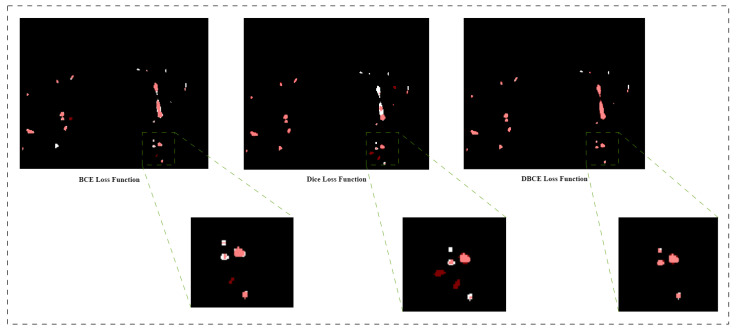
Comparison between BCE Loss Function, Dice Loss Function, and DBCE Loss Function for Segmenting the Fine Details in Pulmonary Artery Segmentation.

Figure 6 illustrates the comparison between segmenting fine details in the pulmonary artery by different loss functions on exemplary testing patches. Ground truth is represented by white color, whereas segmented results are depicted by red color, and the overlap between ground truth and segmented results is shown in pink color. In the case of fine details, the BCE loss function detected more vessels compared to the Dice loss function. The BCE loss function as a local loss function is weighted towards the majority class (background), while the Dice loss function deals with the imbalance datasets, the Dice loss function can have a high variance across batches. The combination of these two loss functions can assist the model in optimizing training at the pixel level along with the image level. It is observed that the pulmonary artery segmented by hybrid loss function has the minimum misclassified voxels and, the segmented contours are closer to the ground contours.

A comparison of different loss functions using the proposed methodology is described in Table 3. It is observed from the table that hybrid loss function has the minimum HD95 and maximum DSC.

In the proposed study, the achieved average values of DSC and HD95 are 0.8775 and 4.2624 mm, respectively. Moreover, Table 4 compares the proposed method with 3D U-Net and the teams that acquired the top three positions in Parse 2022, the pulmonary artery segmentation challenge, their scored positions and the results are available on the website [31]. During the challenge, the results were evaluated on a different test set which is not publicly available.

**Figure 7:**
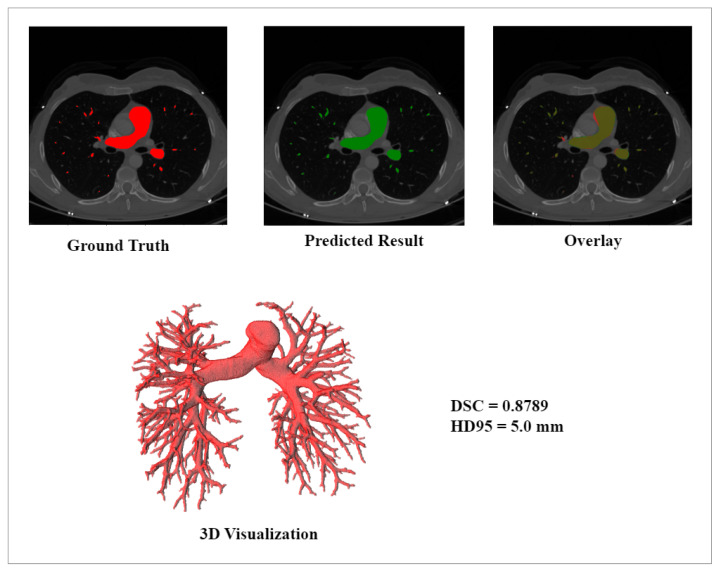
Comparison between Ground Truth and Exemplary Segmented Results with DRU-Net on MICCAI Dataset.

Figure 7 illustrates the ground truth, and predicted result, and overlap between the ground truth and the predicted results, and includes 3D visualizations of the exemplary sample with DSC 0.8789 and HD95 5.0 mm.

## 4. Discussion

Automated segmentation of the pulmonary artery is an essential requirement for medical image analysis. To segment the pulmonary artery, DRU-Net is proposed, which integrates the advantages of dense connection, residual blocks, and U-Net. The features are computed by the combination of residual blocks, encoders, and decoders. In the network, each layer receives the computed features of all previous layers, which helps to improve the flow of information throughout the network.

For the training of the DRU-Net, patches are extracted to accurately segment the pulmonary artery. A patch-based method divides the volume into different patches which generates more data.

The proposed study is evaluated using two metrics, which are the Dice Similarity Coefficient (DSC) and the Hausdorff Distance (HD95). The HD95 contains information on the boundary difference, and the DSC provides details on how much of the generated result overlaps with the ground truth.

In this study, a hybrid loss function is also proposed to segment the pulmonary artery from CT data. Different loss functions are compared using the proposed methodology; results indicate that the hybrid loss function segmented small vessels with greater precision.

Furthermore, the evaluated results are compared with the top three results obtained in MICCAI, PARSE 2022 pulmonary artery segmentation challenge [31]. The top three teams have achieved average DSC 0.7969, 0.7968, and 0.7980, respectively, and the average HD95 attained by the top these teams are 5.2607 mm, 4.7538 mm, and 5.3612 mm. The proposed method has achieved an average DSC of 0.8775 and HD95 of 4.2624 mm. It is observed that DRU-Net was able to correctly segment the aorta and thin branches. The proposed technique will help clinical experts diagnose and plan surgery in case of pulmonary artery hypertension depending on the shape, location, and size of the artery.

## 5. Conclusions

In this article, a DRU-Net is proposed for the segmentation of the pulmonary arteries. The dataset is divided and preprocessed into training, validation, and testing set. In the preprocessing stage, CT scans are enhanced using clipping intensities and random patches are extracted for the training of the DRU-Net. Furthermore, the DRU-Net is trained using 10FCV for segmentation of the pulmonary artery. Additionally, the DBCE hybrid loss function is implemented to segment the fine details. The results achieved in this study are compared with the MICCAI, Parse 2022 pulmonary artery challenge, which demonstrated that our proposed method is effective in segmenting the pulmonary artery.

For technical contributions, our study presents two main ideas:

1. The dense residual network is designed and consists of residual blocks, dense connections, and a U-Net structure. The proposed network addressed the challenges of the vanishing gradient problem and integrated the feature reuse property in the deep neural network.
2. The hybrid loss function combining dice loss and binary cross entropy is introduced to precisely segment fine details in the pulmonary artery.

The proposed methodology employs the following advantages: dense skip connections allow for information flow throughout the network. Due to this, DRU-Net extracts spatial relationships and fine-grained details for segmentation of the pulmonary artery. The residual blocks address the vanishing gradient problem, making it easier for the model to learn and optimize the parameters. It leads to faster convergence and better segmentation. The limitations of the proposed method are: DRU-Net has a deep architecture with a high number of learnable parameters, and it can be computationally costly and require powerful GPUs. To obtain significant segmentation performance, DRU-Net may require a large training data set. Acquiring the diverse dataset with annotations is challenging and time-consuming.

In future work, the pulmonary vein will also be segmented with the pulmonary artery. Segmentation of the pulmonary vein will provide an in-depth view of the entire pulmonary vasculature, allowing for a deeper analysis of lung function and related diseases.

## Figures and Tables

**Table 1:** Dataset Information.

Samples	Age	Gender (F/M)	Pixel Spacing (mm)	Slice Nums
100	29–71 (56.3)	79/21	0.5039–0.9238 (0.6740)	228–390 (303)

**Table 2:** Results of 10FCV for Pulmonary Artery Segmentation.

Folds	DSC	HD95 (mm)
Fold 1	0.8841	4.7503
Fold 2	0.8784	3.7708
Fold 3	0.8762	3.7247
Fold 4	0.8723	3.5432
Fold 5	0.8812	5.2830
Fold 6	0.8720	4.8999
Fold 7	0.8827	4.2403
Fold 8	0.8773	4.1121
Fold 9	0.8785	4.022
Fold 10	0.8730	4.2786

**Table 3:** Comparison of Different Loss Functions.

Loss Functions	DSC	HD95 (mm)
Dice Loss Function	0.8770	4.3666
Binary Cross Entropy (BCE) Loss Function	0.8765	5.5597
Hybrid Loss Function	0.8775	4.2624

**Table 4:** Evaluation comparison with Parse 2022 Pulmonary Artery Segmentation Challenge teams.

Teams	DSC	HD95 (mm)
Infervision medical technology co. Ltd. [31]	0.7969	5.2607
SenseTime China [31]	0.7968	4.7538
Beijing Institute of Technology [31]	0.7980	5.3612
3D U-Net	0.8313	11.6697
DRU-Net (Proposed Method)	0.8775	4.2624

## Data Availability

Pulmonary artery scans used in this study can be accessed via https://parse2022.grand-challenge.org/Dataset/ (9 November 2022) with data usage agreement.
